# Demographic and Clinical Characteristics of Patients Treated With Lecanemab Stratified by Amyloid-Related Imaging Abnormality Status

**DOI:** 10.7759/cureus.114472

**Published:** 2026-08-13

**Authors:** Saurabh Rohatgi, Christina M Younan, Rafael E Martinez Imbett, Jeremy N Ford, Matthew Wleklinski, Shenghua Zhu, Javier Romero

**Affiliations:** 1 Radiology, Massachusetts General Hospital, Harvard Medical School, Boston, USA; 2 Neuroradiology, Massachusetts General Hospital, Harvard Medical School, Boston, USA

**Keywords:** alzheimer’s disease, amyloid, amyloid-related imaging abnormalities, demographic, lecanemab

## Abstract

Background

Amyloid-related imaging abnormalities (ARIAs) are among the most important safety considerations during lecanemab treatment for Alzheimer’s disease (AD). Although *APOE *ε4 carrier status is a recognized ARIA risk factor, the relationship between ARIAs and routine demographic and clinical characteristics in real-world practice remains incompletely characterized. This study evaluated demographic, vascular comorbidity, and *APOE* genotype characteristics among patients treated with lecanemab, stratified by ARIA status.

Methodology

We conducted a retrospective cohort study of patients with AD treated with lecanemab at a single academic institution from December 4, 2023, to October 28, 2025. Of 324 identified patients, 318 were included in the primary analysis. Demographic and clinical variables were extracted from the electronic health record. ARIA status and subtype were determined from clinical MRI interpretations by board-certified neuroradiologists. Patients were stratified by presence and type of ARIA development during treatment.

Results

Among 318 included patients, 98 (30.8%) developed ARIA and 220 (69.2%) did not. Among ARIA-positive patients, nine (9.2%) had ARIA-E only, 50 (51.0%) had ARIA-H only, and 39 (39.8%) had mixed ARIA-E/H. Age was similar between ARIA-positive and ARIA-negative patients (74.4 vs. 73.6 years; p = 0.437). ARIAs occurred in 33.9% of female patients and 26.8% of male patients (p = 0.218), and in 52.9% of non-White patients and 29.6% of White non-Hispanic patients (p = 0.078). Hypertension, hyperlipidemia, and diabetes were not associated with ARIA. *APOE *ε4 carriers had a higher incidence of ARIA than non-carriers (35.3% vs. 22.8%; p = 0.029), although differences across individual *APOE* genotypes did not reach statistical significance (p = 0.135).

Conclusions

In this real-world cohort of patients treated with lecanemab, ARIAs occurred in nearly one-third of patients. *APOE *ε4 carrier status was the only evaluated demographic, clinical, or genetic variable significantly associated with ARIAs. Sex- and race/ethnicity-related differences were exploratory and require validation in larger, more diverse prospective cohorts. These findings support genotype-informed ARIA surveillance while emphasizing the need for broader representation in studies of anti-amyloid therapy safety.

## Introduction

Alzheimer’s disease (AD) is a progressive neurodegenerative disorder characterized pathologically by extracellular β-amyloid (Aβ) deposition, intracellular tau aggregation, synaptic dysfunction, and neurodegeneration. Anti-amyloid immunotherapies have demonstrated clinical efficacy in early AD and represent an important disease-modifying therapeutic option [1,2]. As clinical use of anti-amyloid therapy expands, treatment safety and imaging surveillance have become central components of patient management.

Amyloid-related imaging abnormalities (ARIAs) are recognized adverse imaging findings associated with anti-amyloid monoclonal antibody therapy. ARIA is generally categorized as ARIA-E, reflecting vasogenic edema or sulcal effusion, and ARIA-H, reflecting cerebral microhemorrhage or superficial siderosis [3-5]. Most ARIA events are asymptomatic, but symptomatic and severe cases may occur, particularly in patients with higher-risk clinical or genetic profiles. Therefore, understanding the factors associated with ARIA is essential for patient counseling, treatment selection, and individualized monitoring strategies.

*APOE ε4* is a major genetic risk factor for AD and has also been associated with increased ARIA risk during anti-amyloid therapy [4,6]. However, the extent to which routine demographic variables and vascular comorbidities contribute to ARIA risk in real-world lecanemab practice remains uncertain. This study evaluated demographic, clinical, and *APOE* genotype characteristics in a cohort of patients receiving lecanemab and compared these characteristics by ARIA status.

## Materials and methods

Study design and population

This single-center, retrospective, observational cohort study was conducted at a tertiary academic medical center in Boston, Massachusetts, United States. The study period extended from December 4, 2023, through October 28, 2025 (approximately 23 months) and included patients receiving lecanemab as part of routine clinical care. This retrospective study was approved by the Mass General Brigham Institutional Review Board (IRB) with a waiver of informed consent (approval number: 2024P000596).

A consecutive census sampling approach was used. All patients with AD who received lecanemab during the prespecified study period were screened rather than selecting a random sample. Patients were included when they had (1) a clinical diagnosis of AD for which lecanemab was administered, (2) treatment during the study period, (3) baseline demographic and clinical information available in the electronic health record, and (4) treatment-surveillance MRI information sufficient to determine ARIA status. Patients were excluded when ARIA status could not be determined from the available surveillance imaging or when essential baseline data were incomplete. A total of 324 patients were screened, of whom six were excluded because of incomplete analyzable ARIA status or baseline data, leaving 318 patients in the final analytic cohort.

Clinical and demographic data were extracted from the electronic medical record. Demographic variables included age, sex, and combined race/ethnicity. Race and ethnicity were grouped as non-Hispanic White and non-White to reduce sparse-cell instability in subgroup comparisons. The non-White group included Black or African American, Asian, Hispanic, and Native Hawaiian or Other Pacific Islander patients. Clinical variables included hypertension, hyperlipidemia, diabetes, and *APOE* genotype. Patients with at least one ε4 allele were classified as ε4 carriers.

MRI acquisition and ARIA assessment

MRI examinations were performed using 3-T scanners. A standardized dementia MRI protocol was applied across field strengths and scanner vendors to maintain consistency in image acquisition and clinical interpretation. The protocol included three-dimensional (3D) T2-weighted fluid-attenuated inversion recovery (FLAIR), 2D T2*-weighted gradient-recalled echo (GRE), diffusion-weighted imaging (DWI), 3D T1-weighted anatomic imaging, and multiecho 3D susceptibility-weighted imaging (SWI). Both GRE and SWI were available for assessment of ARIA-H, including cerebral microhemorrhages and superficial siderosis, whereas FLAIR was used for assessment of ARIA-E.

ARIA status was determined from clinical MRI reports interpreted by board-certified neuroradiologists. ARIA subtype was recorded as ARIA-E only, ARIA-H only, or mixed ARIA-E/H. Patients were stratified as ARIA-positive if any ARIA event was documented during lecanemab treatment and ARIA-negative if no ARIA event was documented during available treatment surveillance.

Statistical analysis

Continuous variables are reported as means. Categorical variables are reported as counts and percentages. Age was summarized using the mean and compared between ARIA-positive and ARIA-negative groups using an independent-samples t-test. Categorical variables were summarized as counts and percentages and compared using chi-square tests; Yates’ continuity correction was applied to 2 × 2 contingency tables. The five-category *APOE* genotype comparison was assessed using a Pearson chi-square test. All tests were two-sided, and a p-value <0.05 was considered nominally statistically significant. Because the analyses were exploratory, no adjustment for multiple comparisons was performed.

## Results

ARIA incidence

**Figure 1:**
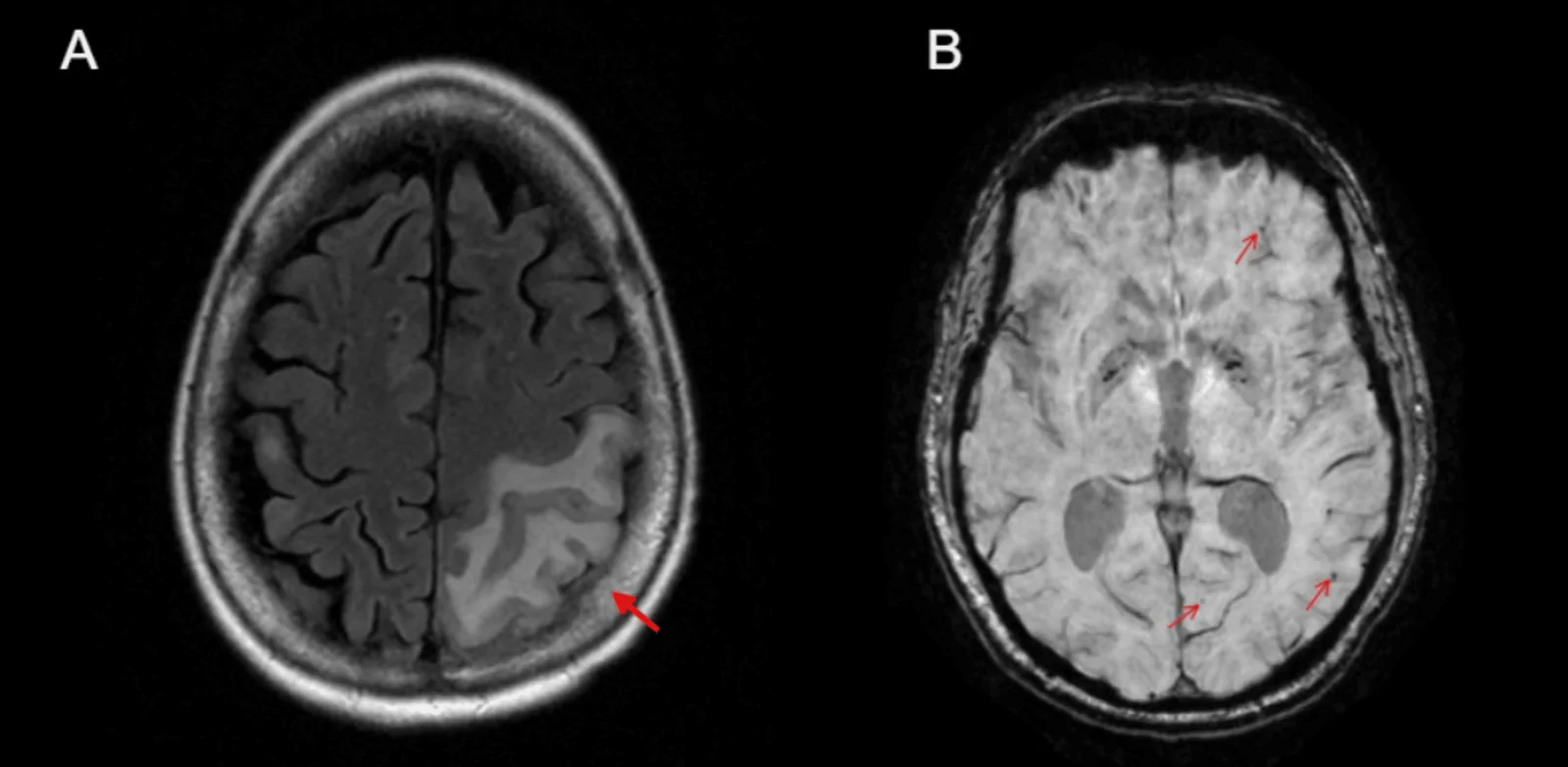
Representative ARIA-E and ARIA-H findings in patients treated with lecanemab. Representative amyloid-related imaging abnormalities (ARIA) in two lecanemab-treated patients. (A) Axial fluid-attenuated inversion recovery (FLAIR) image in a 76-year-old woman shows left parietal vasogenic edema/sulcal effusion (arrow), consistent with ARIA-E. (B) Axial susceptibility-weighted imaging (SWI) image in a 77-year-old man shows multiple punctate susceptibility foci (arrows), consistent with ARIA-H microhemorrhages.

Among 318 included patients treated with lecanemab, 98 (30.8%) developed ARIA and 220 (69.2%) did not. Among the 98 ARIA-positive patients, nine (9.2%) had ARIA-E only, 50 (51.0%) had ARIA-H only, and 39 (39.8%) had mixed ARIA-E/H (Figure 1).

Incidence of ARIA by demographic, clinical, and *APOE* genotype characteristics is shown in Table 1.

**Table 1:** Incidence of ARIA by demographic, clinical, and APOE genotype characteristics. Data are presented as n (%) unless otherwise indicated; percentages are calculated within each ARIA-status column. Hyperlipidemia status was available for 317 patients (97 ARIA-positive and 220 ARIA-negative), and percentages for this variable use nonmissing denominators. Non-White category included Hispanic, Black or African American, Asian, and Native Hawaiian or Other Pacific Islander patients. *: Statistically significant. ARIA = amyloid-related imaging abnormalities; APOE = apolipoprotein E

Characteristic	Total, n	ARIA-positive	ARIA-negative	Test statistic (df)	Test statistic value	P-value
Age (in years)	318	74.4	73.6	t(316)	0.78	0.437
Sex: Female	180	61 (62.2%)	119 (54.1%)	χ²(1)	1.52	0.218
Sex: Male	138	37 (37.8%)	101 (45.9%)			
Race/Ethnicity: Non-White	17	9 (9.2%)	8 (3.6%)	χ²(1)	3.10	0.078
Race/Ethnicity: Non-Hispanic White	301	89 (90.8%)	212 (96.4%)			
Hypertension: Yes	169	51 (52.0%)	118 (53.6%)	χ²(1)	0.02	0.887
Hypertension: No	149	47 (48.0%)	102 (46.4%)			
Hyperlipidemia: Yes	231	73 (75.3%)	158 (71.8%)	χ²(1)	0.25	0.619
Hyperlipidemia: No	86	24 (24.7%)	62 (28.2%)			
Diabetes: Yes	32	11 (11.2%)	21 (9.5%)	χ²(1)	0.07	0.797
Diabetes: No	286	87 (88.8%)	199 (90.5%)			
*APOE* genotype: ε2/ε3	15	3 (3.1%)	12 (5.5%)	χ²(4)	7.01	0.135
*APOE* genotype: ε3/ε3	99	23 (23.5%)	76 (34.5%)			
*APOE* genotype: ε3/ε4	164	56 (57.1%)	108 (49.1%)			
*APOE* genotype: ε4/ε4	37	14 (14.3%)	23 (10.5%)			
*APOE* genotype: ε2/ε4	3	2 (2.0%)	1 (0.5%)			
*APOE* ε4 status: Non-carrier	114	26 (26.5%)	88 (40.0%)	χ²(1)	4.78	0.029*
*APOE* ε4 status: Carrier	204	72 (73.5%)	132 (60.0%)			

Demographic characteristics

**Figure 2:**
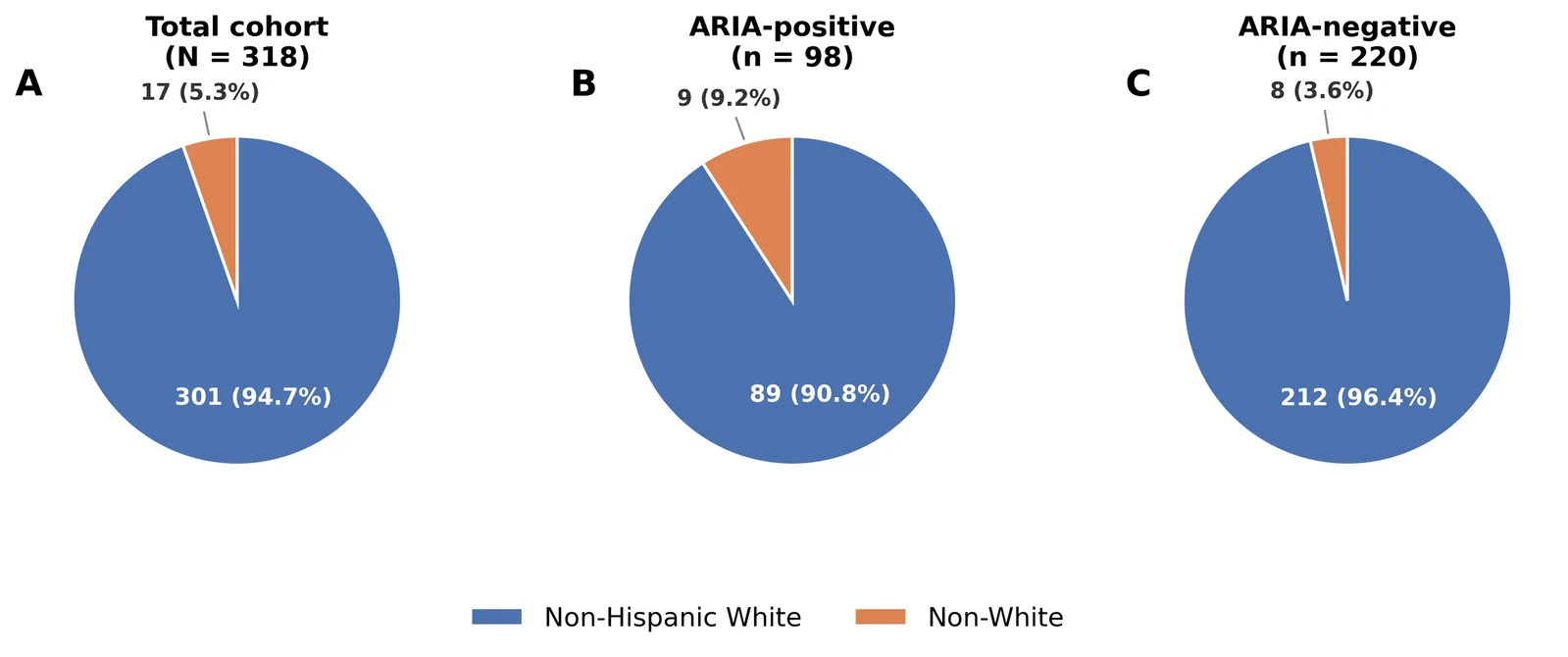
Race/Ethnicity distribution across the cohort and by ARIA status. Pie charts show the proportion of non-Hispanic White and non-White patients in (A) the total cohort (N = 318), (B) ARIA-positive patients (n = 98), and (C) ARIA-negative patients (n = 220). The non-White category included Hispanic, Black or African American, Asian, and Native Hawaiian or Other Pacific Islander patients. ARIA = amyloid-related imaging abnormalities

The mean age of patients who developed ARIA was 74.4 years, compared with 73.6 years among patients who did not develop ARIA (p = 0.437). ARIA occurred in 33.9% (61/180) of female patients and 26.8% (37/138) of male patients (p = 0.218). In the combined race/ethnicity analysis, 52.9% (9/17) of non-White patients developed ARIA compared with 29.6% (89/301) of non-Hispanic White patients (p = 0.078). The race/ethnicity distribution across the total cohort and by ARIA status is shown in Figure 2.

Clinical characteristics

Vascular comorbidities were not associated with ARIA development. ARIA occurred in 30.2% (51/169) of patients with hypertension compared with 31.5% (47/149) of patients without hypertension (p = 0.887). Among patients with hyperlipidemia, 31.6% (73/231) developed ARIA compared with 27.9% (24/86) of patients without hyperlipidemia (p = 0.619). ARIA developed in 34.4% (11/32) of patients with diabetes and 30.4% (87/286) of patients without diabetes (p = 0.797).

Genetic characteristics

When analyzed by specific *APOE* genotype, ARIA developed in 20.0% (3/15) of ε2/ε3 patients, 23.2% (23/99) of ε3/ε3 patients, 34.1% (56/164) of ε3/ε4 patients, 37.8% (14/37) of ε4/ε4 patients, and 66.7% (2/3) of ε2/ε4 patients (p = 0.135). When *APOE* genotype was dichotomized by ε4 carrier status, 35.3% (72/204) of ε4 carriers developed ARIA compared with 22.8% (26/114) of non-carriers (p = 0.029). APOE ε4 carrier status was the only evaluated variable that reached statistical significance.

## Discussion

This retrospective cohort study evaluated demographic, clinical, and *APOE* genotype characteristics among patients with AD treated with lecanemab. ARIAs occurred in 30.8% of included patients, higher than the 21.3% ARIA rate reported in the pivotal Clarity AD trial [7]. This difference may reflect real-world patient heterogeneity, variations in imaging surveillance, and the broader implementation of lecanemab outside controlled trial settings [8]. In our cohort, standardized surveillance MRI included both GRE and SWI for assessment of hemorrhagic manifestations of ARIA. Differences in susceptibility-sensitive imaging acquisition and interpretation may influence detection of cerebral microhemorrhages and superficial siderosis and could therefore contribute to differences in observed ARIA-H rates across studies. However, given differences in study design and the absence of centralized blinded imaging adjudication in the present study, the higher observed ARIA incidence cannot be attributed specifically to MRI sequence selection.

The distribution of ARIA subtypes was dominated by ARIA-H only and mixed ARIA-E/H. In Clarity AD, ARIA-H was more frequent than ARIA-E, and co-occurrence of edema/effusion and hemorrhagic findings was also observed [7]. The relatively high proportion of mixed ARIA-E/H in the present cohort may suggest overlapping vascular and amyloid-related mechanisms, although this interpretation requires confirmation with standardized imaging review and prospective adjudication [6,7].

*APOE* ε4 carrier status was significantly associated with ARIA development. ARIA occurred in 35.3% of ε4 carriers compared with 22.8% of non-carriers. Although the genotype-level comparison did not reach statistical significance, ARIA incidence was numerically higher in ε3/ε4 and ε4/ε4 patients than in ε2/ε3 and ε3/ε3 patients. These findings are consistent with prior reports linking *APOE* ε4 to greater amyloid burden, cerebral amyloid angiopathy, blood-brain barrier vulnerability, and ARIA risk during anti-amyloid therapy [6]. However, because the present analysis did not adjust the *APOE* ε4 association for potential demographic and vascular confounders, an independent effect of *APOE* ε4 cannot be established from this study.

Age, sex, hypertension, hyperlipidemia, and diabetes were not significantly associated with ARIA in this cohort. ARIA occurred more often in female patients than in male patients, but this difference did not reach statistical significance. Prior studies of anti-amyloid therapy have not consistently shown sex-based differences in ARIA; however, biologic interactions among sex, *APOE* genotype, amyloid deposition, and cognitive outcomes have been described in AD and may warrant further ARIA-specific evaluation [9,10]. Similarly, vascular comorbidities are biologically plausible contributors to ARIA susceptibility but have not shown consistent associations across clinical studies, potentially because of differences in medication control, exclusion criteria, and sample size [10,11].

A non-significant trend toward higher ARIA incidence was observed among non-White patients compared with non-Hispanic White patients. This finding should be interpreted with substantial caution because the non-White subgroup was small and combined multiple racial and ethnic groups into a single analytic category. Nonetheless, the observation highlights an important limitation in the current evidence base for anti-amyloid therapies. Racial and ethnic differences in dementia diagnosis, amyloid biomarker positivity, and *APOE*-associated risk have been described, and underrepresentation of minoritized populations in AD therapeutic trials remains a major challenge [12-17]. Larger and more diverse cohorts will be needed to determine whether race, ethnicity, ancestry, access to care, comorbidity burden, or imaging surveillance patterns modify ARIA risk.

This study has several limitations. The retrospective design limits causal inference and may introduce documentation, referral, and surveillance biases. ARIA classification was based on clinical radiology reports rather than on centralized, blinded adjudication or quantitative imaging assessment. The cohort was drawn from a single academic institution, and the small number of non-White patients limited the precision of race/ethnicity subgroup analyses. In addition, the current analysis focused on baseline demographic, clinical, and *APOE* characteristics. Future models should incorporate treatment exposure, MRI field strength and sequence parameters, baseline microhemorrhage burden, white matter disease severity, amyloid burden, antithrombotic exposure, and longitudinal surveillance frequency. In addition, the association between *APOE* ε4 carrier status and ARIAs was based on unadjusted analyses. Demographic and vascular characteristics were not evaluated according to *APOE* ε4 carrier status, and residual confounding cannot therefore be excluded.

Despite these limitations, the study provides real-world evidence that complements clinical trial data. Most routine demographic and vascular comorbidity variables were not significantly associated with ARIA, whereas *APOE* ε4 carrier status showed a significant unadjusted association with higher ARIA incidence. These findings support continued incorporation of APOE genotype into patient counseling and surveillance planning, while emphasizing that demographic trends should not be overinterpreted without larger, more representative datasets.

## Conclusions

In this real-world cohort of patients treated with lecanemab, ARIAs occurred in a substantial proportion of patients during treatment surveillance. *APOE* ε4 carrier status was the only evaluated variable that showed a statistically significant association with ARIA development, whereas age, sex, vascular comorbidities, and combined race/ethnicity categories did not reach statistical significance. These findings support genotype-informed counseling and monitoring for patients receiving lecanemab. At the same time, the exploratory differences observed across sex and race/ethnicity subgroups underscore the need for prospective, multicenter studies with standardized imaging adjudication and broader demographic diversity. Such studies will be essential to refine ARIA risk stratification and to support the safe, equitable implementation of anti-amyloid therapies in routine clinical practice.
